# Clinical course in a patient with myopathic VLCAD deficiency during pregnancy with an affected baby

**DOI:** 10.1002/jmd2.12061

**Published:** 2019-07-17

**Authors:** Kenji Yamada, Keiichi Matsubara, Yuko Matsubara, Asami Watanabe, Sanae Kawakami, Fumihiro Ochi, Kozue Kuwabara, Yuichi Mushimoto, Hironori Kobayashi, Yuki Hasegawa, Seiji Fukuda, Seiji Yamaguchi, Takeshi Taketani

**Affiliations:** ^1^ Department of Pediatrics Shimane University Faculty of Medicine Izumo Shimane Japan; ^2^ Department of Obstetrics and Gynecology Ehime University School of Medicine Toon Ehime Japan; ^3^ Department of Pediatrics Yawatahama City General Hospital Yawatahama Ehime Japan; ^4^ Department of Pediatrics Ehime University Graduate School of Medicine Toon Ehime Japan; ^5^ Department of Pediatrics, Graduate School of Medical Sciences Kyushu University Higashi‐ku Fukuoka Japan

**Keywords:** familial screening, placenta, pregnancy, ritodrine, rhabdomyolysis, very long‐chain acyl‐CoA dehydrogenase deficiency

## Abstract

Very long‐chain acyl‐CoA dehydrogenase (VLCAD) deficiency is an autosomal recessive mitochondrial fatty acid oxidation disorder that manifests in three clinical forms: (a) severe, (b) milder, and (c) myopathic. Patients with the myopathic form present intermittent muscular symptoms such as myalgia, muscle weakness, and rhabdomyolysis during adolescence or adulthood. Here, the clinical symptoms and serum creatine kinase (CK) levels of a pregnant 31‐year‐old woman with the myopathic form of VLCAD deficiency were reduced during pregnancy. Clinical symptoms rarely appeared during pregnancy, although she had sometimes suffered from muscular symptoms before pregnancy. When ritodrine was administered for threatened premature labor at 35 weeks of gestation, her CK level was elevated to over 3900 IU/L. She delivered a full‐term baby via cesarean section but suffered from muscle weakness with elevated CK levels soon after delivery. It has been reported that an unaffected placenta and fetus can improve maternal β‐oxidation during pregnancy. However, in our case, the baby was also affected by VLCAD deficiency. These suggest that the clinical symptoms of a woman with VLCAD deficiency might be reduced during pregnancy even if the fetus is affected with VLCAD deficiency.

## 1 INTRODUCTION

Very long‐chain acyl‐CoA dehydrogenase (VLCAD) deficiency (OMIM 201475) is a long‐chain fatty acid oxidation disorder.^1^ VLCAD deficiency shows an autosomal recessive inheritance pattern, and its detection incidence in Japanese newborn screening is estimated to be approximately 1:93 000 births.^2^ VLCAD deficiency is roughly classified into three clinical phenotypes: (a) an early‐onset severe form (severe form), (b) a childhood‐onset milder form (milder form), and (c) a late‐onset myopathic form (myopathic form).^3^ Patients with the milder or myopathic form of VLCAD deficiency exhibit occasional clinical symptoms, such as hypoketotic hypoglycemia, liver dysfunction, myalgia, hypotonia, or rhabdomyolysis, which are often triggered by prolonged fasting, infectious illnesses, or excessive physical exercise.

A few reports have stated that the symptoms of women with VLCAD deficiency often improve during pregnancy, probably due to compensation of maternal β‐oxidation by the unaffected placenta and fetus.^4^ Here, we present a case in which both the mother and the baby were affected by the milder form of VLCAD deficiency, and the mother's symptoms improved during pregnancy.

## 2 CASE REPORT

**Figure 1:**
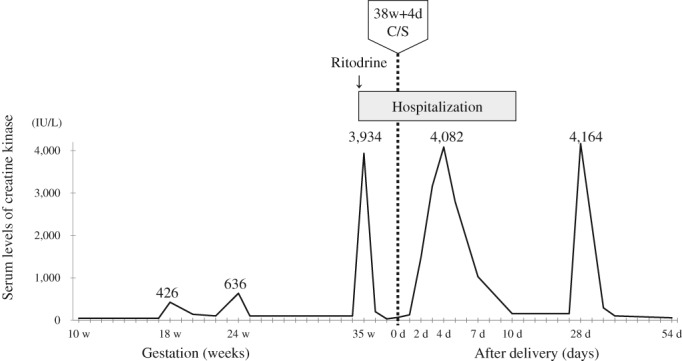
Serum creatine kinase levels and clinical course from pregnancy to delivery. C/S, cesarean section; d, day; w, week

The patient was a 31‐year‐old woman without consanguinity in her family history. This patient had presented with myalgia after exercise at preschool age. Resting and/or glucose infusion improved her muscular symptoms during these episodes. At 26 years of age, a muscle biopsy and immunostaining analysis revealed a lack of reaction for VLCAD protein in her muscle tissues. Eventually, a homozygous p.K382Q mutation in *ACADVL* was identified after delivery. Because her main symptoms were myalgia and muscular weakness, she was diagnosed with the myopathic form of VLCAD deficiency despite childhood onset. Before pregnancy, the woman suffered from muscle weakness and myalgia four or five times per year but rarely visited the hospital because she opted for self‐treatment. At 31 years of age, she underwent in vitro fertilization and embryo transfer and then became pregnant. Although the patient had hyperemesis gravidarum around the first trimester of pregnancy, only mild myalgia was observed. During pregnancy, she frequently drank juices to prevent metabolic attacks. A few episodes, such as no subjective symptoms with mild elevations in creatine kinase (CK) levels at the 18th week of gestation and mild fatigue with elevated CK at the 24th week, were observed (Figure 1). At 35 weeks and 2 days of gestation, the threat of premature labor emerged. In an effort to prevent premature labor, hospitalization with rest and ritodrine administration was initiated. However, because of an elevation in CK levels (3934 IU/L; normal range, 45‐226) and resolution of uterine contractions, ritodrine treatment was immediately ceased. At 38 weeks and 4 days of gestation, the woman successfully delivered a girl whose birth weight was 2894 g via cesarean section without maternal complications. Beginning 2 days after delivery, muscle weakness coincident with elevation in CK levels recurred.

Her baby was hospitalized due to transient tachypnea, but the baby immediately recovered without metabolic attacks. Newborn screening using tandem mass spectrometry was not performed because the family was unwilling. At 4 months of age, the baby was definitively diagnosed with VLCAD deficiency by genetic testing, which showed compound heterozygous p.R229X/p.K382Q mutations in *ACADVL* in the infant and a heterozygous p.R229X mutation in her husband. These mutations were previously reported. Although she was temporarily asymptomatic after diagnosis, she developed myalgia and fatigue two to three times per year since the age of 1 year. However, hypoglycemia attacks have not been noted.

## 3 DISCUSSION

We report a woman with the myopathic form of VLCAD deficiency whose muscular symptoms improved during pregnancy and who had an affected baby with VLCAD deficiency. Similar cases have never been reported. Symptoms in affected pregnant females with VLCAD deficiency were previously reported to improve during pregnancy, suggesting that the non‐affected fetus and placenta can enhance the maternal metabolism of fatty acid oxidation.^4^ Indeed, the muscular symptoms, C14:1 acylcarnitine levels, and CK levels were reduced, particularly during the second and third trimesters, in several pregnant women with VLCAD deficiency who delivered unaffected babies.^4^, ^5^, ^6^ However, in our case, the baby also had VLCAD deficiency. The improvements in muscular symptoms could be attributable to resting and the frequent intake of carbohydrates, but the patient's muscular symptoms recurred soon after delivery, indicating that the affected placenta and fetus also played an important role in improving maternal β‐oxidation during pregnancy. Because fatty acid oxidation enzymes, including VLCAD, are highly expressed in the human placenta,^7^ quantitative enhancement of these enzymes might contribute to the improvement in maternal β‐oxidation despite mutant VLCAD. Nevertheless, our hypothesis could not be scientifically proven because we had not investigated objective markers, such as her cardiac function or CK and acylcarnitine levels before and after pregnancy, and because we did not collect her fibroblasts or placenta. We were also concerned about the difference in the pregnancy courses of the affected and unaffected fetus, but this woman never became pregnant after this episode. Minimally, our cases suggest that the symptoms of VLCAD deficiency can be improved during pregnancy regardless of whether the baby is affected.

In contrast, muscular symptoms worsened soon after delivery in all previously reported cases. Our patient's clinical course was similar to those of the previous cases. A patient with adult‐onset VLCAD deficiency who developed acute postpartum heart failure due to cardiomyopathy has previously been reported.^8^ Furthermore, Yamamoto et al speculated that hormonal changes during puerperium might represent a risk factor for metabolic crises. Therefore, hospitalization and/or continuous glucose infusion after delivery in puerperant women with fatty acid oxidation disorders, including VLCAD deficiency, may be preferable.

We chose cesarean section as the mode of delivery because we were concerned that the strain and labor of vaginal delivery might worsen the patient's condition. In general, cesarean section for women with VLCAD deficiency should be considered based on (a) obstetric indications or (b) clear maternal or fetal benefit.^4^ Additionally, perioperative fasting can also be a risk factor. However, surgery carries little risk even for patients with VLCAD deficiency when adequate perioperative management is carried out with a minimal fasting period, minimal surgical stress, and appropriate glucose infusion.^9^ It has also been suggested that the combination of epidural anesthesia, medium‐chain triglyceride treatment, and glucose infusion might reduce fatigue during labor.^6^ Although we cannot conclusively determine which mode of delivery is best, cesarean section was safely performed in our case.

Finally, ritodrine might increase CK levels in our case. Ritodrine is a β‐stimulator and can also cause rhabdomyolysis as an adverse effect; caution should be exercised when this drug is administered to pregnant women, such as the one in this case, although the cause is unknown.

## 4 CONCLUSION

The metabolism of fatty acid oxidation can be enhanced even by the affected placenta and fetus, particularly the placenta, in pregnant females with VLCAD deficiency. In women with VLCAD deficiency, pregnancy management should be a concern regardless of whether or not the baby is affected.

## AUTHOR CONTRIBUTIONS

K. Yamada designed the study, wrote the initial draft of the manuscript, and acquired the funding. K.M. and Y.M. were the attending physicians of the mother and initially reported her case as an oral presentation in the local association of The Japan Society of Obstetrics and Gynecology. A.W., S.K., F.O., and K.K. were the attending physicians of the baby and provided the information on the mother and baby. Additionally, they obtained informed consent from the patients. Y.M., H.K., and Y.H. contributed to the analysis and interpretation of the data and revised the manuscript. S.F., S.Y., and T.T. critically revised this draft for important intellectual content and provided the final approval to submit this article.

## CONFLICT OF INTEREST

The authors declare that they have no conflicts of interest.

## INFORMED CONSENT

Informed consent was verbally obtained from the patient, and the content was recorded on her medical chart. Additionally, the submission of this manuscript was approved by the Institutional Review Board of Shimane University.

## ETHICAL APPROVAL

This article does not contain any studies with human or animal subjects performed by any of the authors.
